# Phenotype Sequencing: Identifying the Genes That Cause a Phenotype Directly from Pooled Sequencing of Independent Mutants

**DOI:** 10.1371/journal.pone.0016517

**Published:** 2011-02-18

**Authors:** Marc A. Harper, Zugen Chen, Traci Toy, Iara M. P. Machado, Stanley F. Nelson, James C. Liao, Christopher J. Lee

**Affiliations:** 1 Institute for Genomics and Proteomics, University of California Los Angeles, Los Angeles, California, United States of America; 2 Department of Human Genetics, University of California Los Angeles, Los Angeles, California, United States of America; 3 Department of Chemical Engineering, University of California Los Angeles, Los Angeles, California, United States of America; 4 Department of Chemistry and Biochemistry, University of California Los Angeles, Los Angeles, California, United States of America; 5 Department of Computer Science, University of California Los Angeles, Los Angeles, California, United States of America; 6 Molecular Biology Institute, University of California Los Angeles, Los Angeles, California, United States of America; Duke University, United States of America

## Abstract

Random mutagenesis and phenotype screening provide a powerful method for dissecting microbial functions, but their results can be laborious to analyze experimentally. Each mutant strain may contain 50–100 random mutations, necessitating extensive functional experiments to determine which one causes the selected phenotype. To solve this problem, we propose a “Phenotype Sequencing” approach in which genes causing the phenotype can be identified directly from sequencing of multiple independent mutants. We developed a new computational analysis method showing that 1. causal genes can be identified with high probability from even a modest number of mutant genomes; 2. costs can be cut many-fold compared with a conventional genome sequencing approach via an optimized strategy of library-pooling (multiple strains per library) and tag-pooling (multiple tagged libraries per sequencing lane). We have performed extensive validation experiments on a set of *E. coli* mutants with increased isobutanol biofuel tolerance. We generated a range of sequencing experiments varying from 3 to 32 mutant strains, with pooling on 1 to 3 sequencing lanes. Our statistical analysis of these data (4099 mutations from 32 mutant genomes) successfully identified 3 genes (*acrB, marC, acrA*) that have been independently validated as causing this experimental phenotype. It must be emphasized that our approach reduces mutant sequencing costs enormously. Whereas a conventional genome sequencing experiment would have cost $7,200 in reagents alone, our Phenotype Sequencing design yielded the same information value for only $1200. In fact, our smallest experiments reliably identified *acrB* and *marC* at a cost of only $110–$340.

## Introduction

High-throughput sequencing is a potentially powerful tool for analyzing microbial mutant strains with interesting phenotypes, because it can quickly identify their complete set of mutations. However, unless one has prior knowledge that all or most of the mutations must contribute to the phenotype, these data can be hard to interpret. The fewer the mutations, the more likely it is that a given mutation contributes to the observed phenotypic difference. From this point of view, the easiest cases appear to be strains evolved without artificial mutagenesis, which typically contain only 10–20 mutations per bacterial genome [1] [2] [3] [4] [5] [6], with in some cases as few as 3 mutations per strain [7] [8] or more than 40 [9]. These data present two kinds of problems: how to identify which mutation makes the dominant contribution to the phenotype, and how to filter out mutations that are either neutral or simply not relevant to the desired phenotype. Both kinds of problems may necessitate extensive functional experiments to determine which mutations actually cause the selected phenotype. These problems grow more difficult if mutants are generated via random mutagenesis, since each mutant strain may contain 50 to 100 or more random mutations [10] [11], out of which perhaps only one is responsible for the phenotype.

Simple numerical considerations can illustrate this problem. For the *E. coli* genome (4244 genes), assuming that mutations in ten different genes can give rise to the desired phenotype, the probability of picking one of these genes purely by random chance is about 0.25%. If we generate a mutant strain with the desired phenotype, sequence it, and identify 100 mutated genes, our chances of picking a gene that causes the phenotype rise only nominally, to 1%.

However, if we can obtain multiple independent mutant strains from our phenotype screen, the statistics of independent selection events will quickly help distinguish the true target genes. Even in the worst case (only a single one of the 100 mutations in each strain is required to be in a true target gene, split with equal probability among the ten target genes), the mutation frequency in true target genes (approximately one in ten) is expected to be four times greater than that expected in non-target genes (approximately one in forty). As more mutant strains are sequenced, true target genes are guaranteed by the Law of Large Numbers to rise above the background noise. This suggests the possibility of identifying true target genes automatically, directly from sequencing data. We will refer to this approach as “phenotype sequencing”.

In this paper we present a combination of bioinformatic and experimental analysis of the phenotype sequencing problem. We begin by formulating a mathematical model of phenotype sequencing, analyzing the parameters that determine the likelihood of success. We next present a high-throughput sequencing design optimized for phenotype sequencing. It uses a combination of library-pooling and tag-pooling to reduce the cost of a phenotype sequencing experiment many-fold relative to a standard mutant genome resequencing design, while fully retaining the information needed for identifying the genetic causes of the phenotype. We then demonstrate the method via sequencing of a set of 32 *E. coli* mutants selected for increased isobutanol biofuel tolerance. We show that our phenotype sequencing bioinformatic tools successfully identify a number of genes directly from the sequencing data, and have been validated by independent experiments. Finally, we assess the broad applicability of phenotype sequencing by analyzing its yield vs. cost both experimentally and computationally, in terms of a number of key factors such as mutagenesis density, sequencing error rates, and sequencing cost. These results indicate that phenotype sequencing can become a rapid, inexpensive and automatic method applicable to a wide variety of microbial phenotypes.

## Results

### Mathematical analysis of Phenotype Sequencing

**Figure 1:**
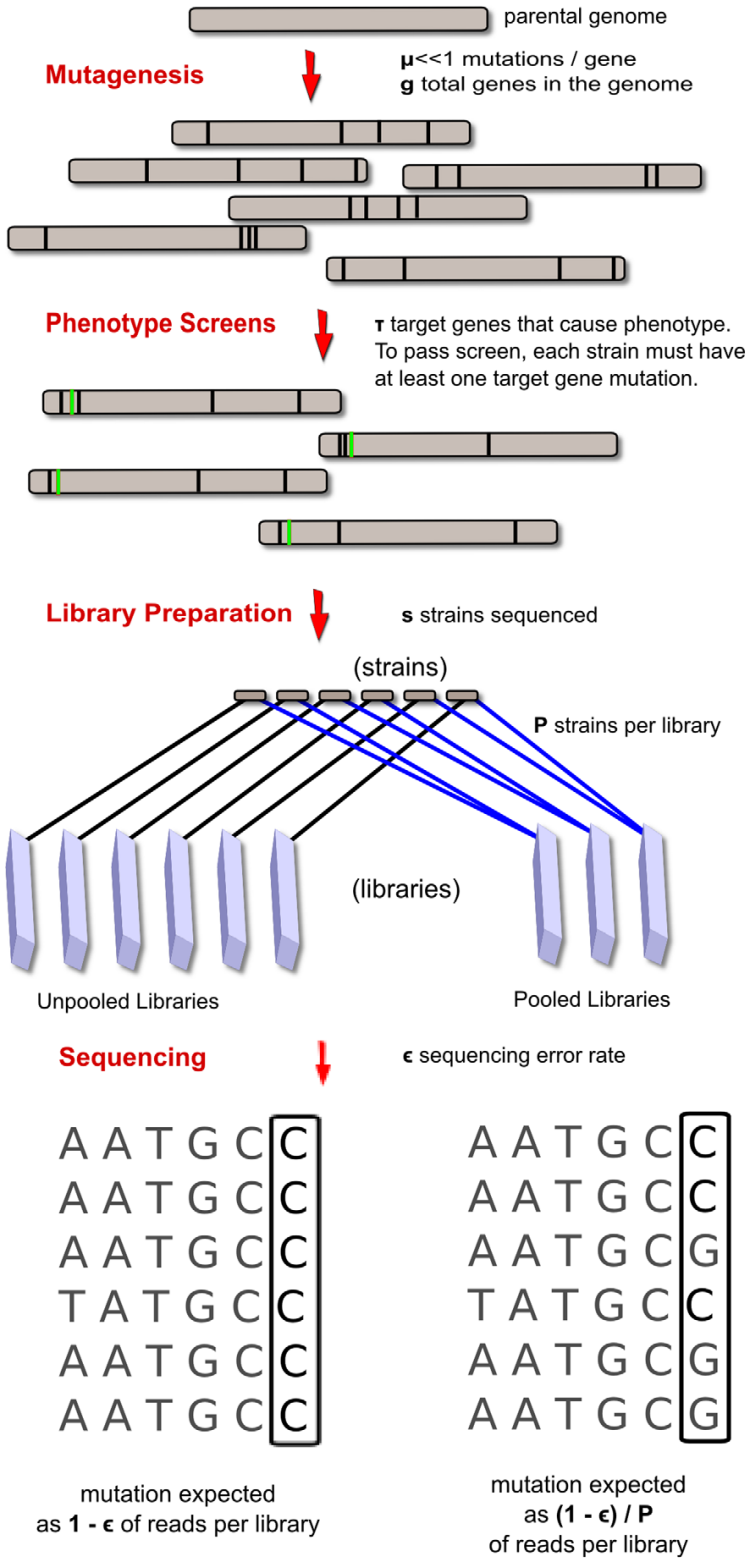
Schematic diagram of phenotype sequencing and key parameters. Overview of phenotype sequencing stages: mutagenesis, screening, and sequencing. Conventional unpooled sequencing of individual strains (left), is contrasted with pooled sequencing of multiple strains per library (right), comparing the expected frequency of observation of a real mutation in each case.

We begin by analyzing the probability of successfully identifying the genetic causes of a phenotype. We have constructed a mathematical model of the phenotype screening and sequencing process (see Materials and Methods for details). This analysis reveals the critical importance of several parameters in the overall process of phenotype sequencing (shown in schematic outline in Fig. 1): the average density of mutations in each mutant strain (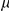); the number of genes where mutations can cause the phenotype (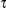; we will refer to these as “target genes”); and the number of independent mutant strains that pass the phenotype screen and are sequenced (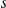). We first analyze the most difficult form of the problem, by adopting the conservative assumptions that only a single target gene mutation is required to produce the phenotype, and that such phenotype-causing mutations are split with equal likelihood among the possible target genes. This poses the challenge of identifying the single causal mutation in a strain out of its 100 or so total mutations. For simplicity, we assume that synonymous mutations will not cause the phenotype, and therefore restrict our analysis to non-synonymous mutations.

We used our mathematical analysis to calculate the average yield of true target genes discovered among the top-scoring hits at a specified false discovery rate (FDR). Concretely, this means we randomly generate 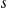 mutant strains with an average density of random mutations 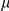, under the requirement that each mutant strain must include at least one mutation in a true target gene. We then score each gene by calculating a p-value based on its total number of mutations in the 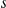 strains (see Materials and Methods), sort the genes by score, and determine the number of true target genes found among the top-scoring genes at an 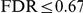 (i.e. out of every three genes reported, at least one must be a true target). To measure the average yield, we repeated this process 1000 times. Thanks to speed optimizations, our *phenoseq* software can model over 600,000 mutant genomes per second on a single core of a 2.5 GHz Core 2 Duo CPU (early 2008 MacBook Pro).

**Figure 2:**
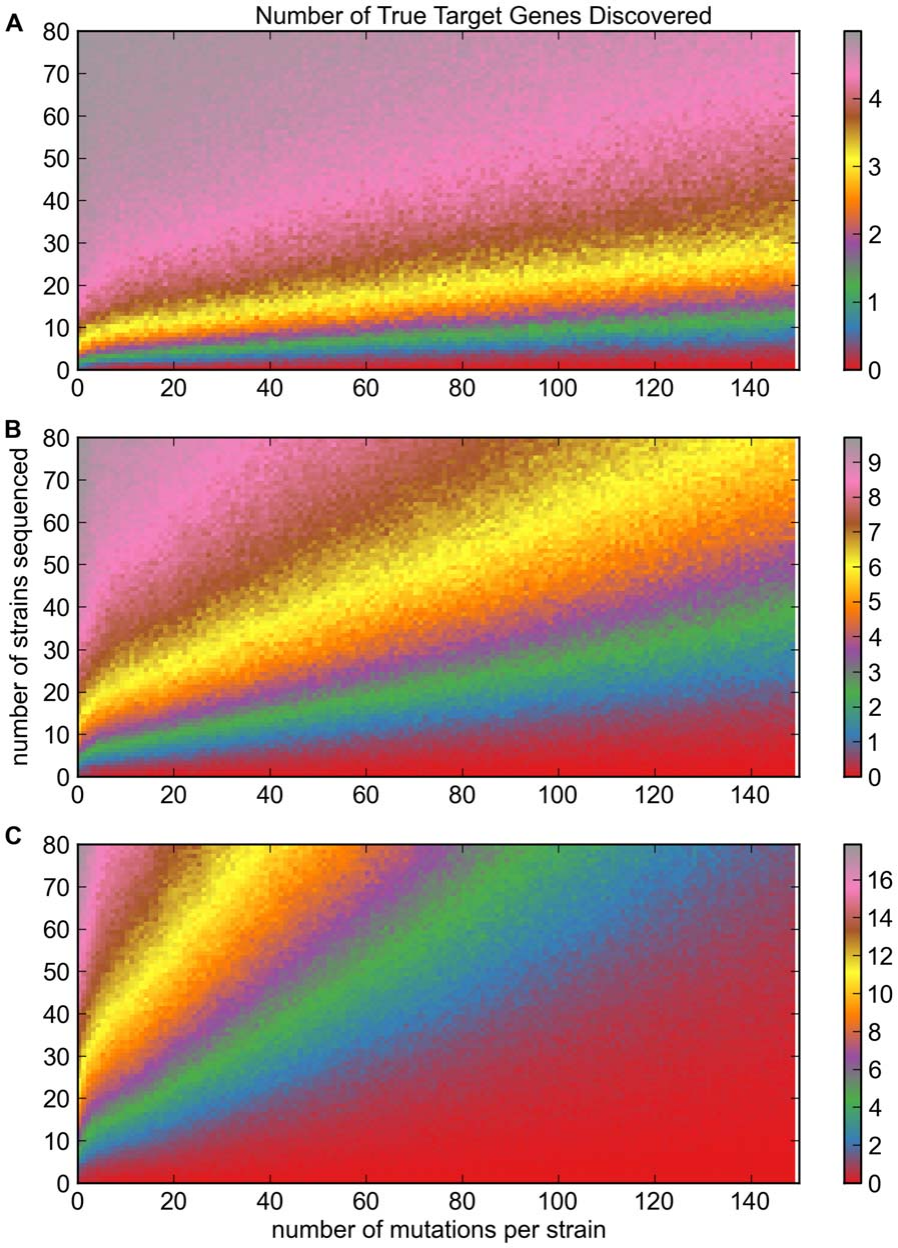
Target discovery yield as a function of mutations per strain and number of strains sequenced. **A**. For five target genes. Gray color (upper-left corner) represents discovery of all 5 targets; red  =  zero targets. **B**. For ten target genes. Gray represents discovery of all 10 targets. **C**. For twenty target genes. Gray represents discovery of all 20 targets.

These results show that phenotype sequencing will work well with a modest number of sequenced strains, even under our most challenging assumptions, for a range of typical target sizes. For example, if the phenotype-causing mutations are split equally among 5 genes (i.e. 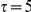), and each mutant strain contains 50–100 total mutations (of which, by random chance, only 30–70 would be non-synonymous), sequencing of 10 strains on average successfully identifies 2 of the five true target genes among the top-scoring genes at an 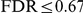 (Fig. 2A). If the phenotypic signal is split over an even larger number of genes, the problem grows harder. For 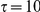 genes, sequencing of 10 strains detects on average 1–2 of the ten true target genes (Fig. 2B). If we sequence 30 strains, the expected yield rises to 4–6 true target genes. For 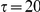 genes, sequencing 30 strains will on average identify 2–4 of the 20 true target genes (Fig. 2C).

These data also show an approximately linear relationship between the number of mutations per strain and the number of strains that must be sequenced to attain a given yield. In all cases, increasing the density of mutagenesis means that the number of strains sequenced must increase proportionately, in order to maintain the same average yield of target gene discovery. This makes intuitive sense: if only one mutation per strain is signal (actually causes the phenotype), then increasing the number of irrelevant mutations per strain will reduce the signal-to-noise ratio. These data indicate that where possible, investigators should reduce the density of mutagenesis to a smaller number of mutations per strain, to maximize the yield of target gene discovery and minimize the number of strains that must be sequenced. They also suggest that naturally evolved mutant strains, which tend to have a smaller number of mutations (typically 10–20 mutations per bacterial genome), will be easier, more successful targets for phenotype sequencing (as always, assuming that it is possible to obtain a sufficient number of independent mutant strains with the desired phenotype).

#### Analysis of Phenotype Sequencing via Pooling

Since phenotype sequencing requires sequencing complete genomes of multiple mutant strains, and is potentially expensive, we wish to optimize the information yield per cost. Ordinarily, mutant genome sequencing is performed by preparing individual DNA libraries and sequencing each library separately. Since the goal of phenotype sequencing is to identify the genes that actually cause the phenotype, we will consider the sequences of the individual mutant strains as merely a means toward this goal, and not an end in themselves. For phenotype sequencing, the key piece of information is just the number of times a gene is independently mutated; the exact sequence of each mutant strain is not needed. From this point of view, we can dramatically reduce costs by pooling multiple mutant strains in two distinct ways: 1. *library-pooling*: mixing equal amounts of DNA from multiple mutant strains into a single library preparation. This sacrifices the ability to reconstruct the exact sequence of each mutant strain, but retains our ability to identify how many distinct mutations occur in each gene; 2. *tag-pooling*: if each library is tagged with a unique DNA sequence, multiple libraries can be combined into a single sequencing lane. Pooling has been shown to be an effective way of reducing costs of population genetics studies (e.g. estimation of population allele frequencies) using next-generation sequencing [12] [13] [14]. Since microbial genomes are small, many copies of a genome can be sequenced in a single lane. For example, an Illumina GA2x sequencing lane with 1500 Mb sequencing capacity can sequence the 4.6 Mb *E. coli* genome at approximately 323x coverage. This is sufficient to sequence five libraries simultaneously at about 65x coverage each.

**Figure 3:**
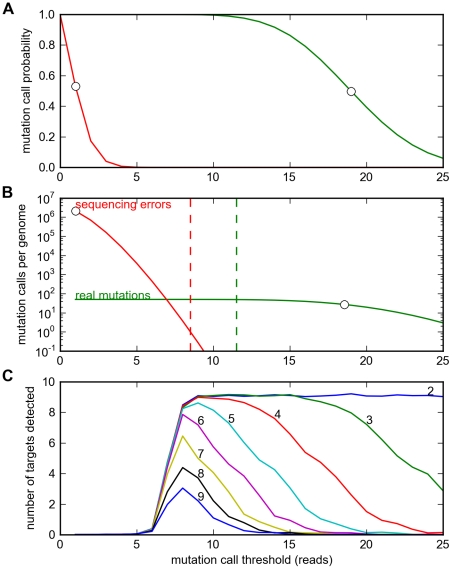
Effects of sequencing error and pooling on average target gene discovery yields. **A**. The probability of reporting a SNP at a single site as a function of the mutation call threshold (read counts) assuming a coverage of c = 75, due either to sequencing error (red), or a real mutation (green), assuming a 1% sequencing error rate and a 25% true mutation fraction (i.e. library-pooling factor of P = 4). Circles indicate the expected mean read counts on each plot. **B**. The expected number of total mutation calls per genome as a function of the mutation call threshold, due either to sequencing error (red), or a real mutation (green), assuming a 4 Mb genome size. The dashed red line indicates the lowest mutation call threshold at which the number of false positive mutation calls falls below one. The dashed green line indicates the maximum mutation call threshold at which the number of false negatives remains less than one. **C**. The average number of true target genes discovered (at an FDR <0.67) as a function of the mutation call threshold, for different library-pooling levels P = 2 to P = 9, assuming sequencing of 80 mutant strains with a mutation density of 50 mutations per genome, and 20 true target genes.

To analyze the effects of pooling, we extended our model of phenotype sequencing to take sequencing error into account. Sequencing error can cause two kinds of problems: *false positives*, i.e. a mutation is reported where none actually exists; *false negatives*, i.e. a real mutation present in the DNA is not reported. False positives make phenotype sequencing much harder by spuriously increasing the apparent mutation density per genome. False negatives can also reduce success rates, by diminishing mutation counts in true target genes. For a given average sequencing coverage level 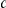, two key parameters determine the false positive and false negative rates: the sequencing error probability 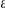 and the number of strains 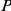 pooled together in one library. For example, for pooling 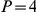 strains, a real mutation in one strain is expected to occur in approximately 25% of the reads that cover that position, much higher than the fraction of a single alternate base expected from sequencing errors at that position (we use a conservative estimate of 1% for this rate). However, in practice we must discriminate these two cases using discrete counts of the number of reads that report an alternate letter. For a single nucleotide site, these two cases are very easy to distinguish (Fig. 3A). For genome-wide analysis the problem is much harder; we wish to keep the total number of false positives (over all 4.6 million sites in the genome) to less than one, while keeping the total number of false negatives over the whole genome also to less than one (Fig. 3B). For a standard coverage level of 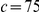, this restricts us to a fairly narrow “ideal zone” for the mutation call threshold. It is evident that if we pooled a much larger number of strains 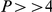, it would simply not be possible to achieve *both* low false positive and low false negative rates.

To analyze this effect, we computed the false positive and negative rates for every possible cutoff, over a wide range of pooling values, and used them to compute the average target gene discovery yield at each point (Fig. 3C). Each yield curve for a given pooling value is bounded on the left by a sharp cutoff value; this occurs because cutoff values that are too low give high false positive rates, quickly reducing the yield to zero. At higher cutoff values, the false positive rate goes to zero, and the yield saturates. However, if the cutoff value approaches 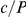, the false negative rate increases gradually, and consequently the yield drops.

These computations show that at their optimal cutoff values, pooling values of 2–5 give almost exactly the same yield as no pooling at all (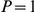). Thus pooling at these levels fully retains the information important for phenotype sequencing while dramatically reducing cost. For example, pooling five strains per library reduces the total cost by a factor of five, with little information loss in terms of the yield of true target genes discovered.

### Experimental Results

Based on these encouraging bioinformatic results, we designed a phenotype sequencing experiment based on library-pooling and tag-pooling. We isolated DNA from 32 mutant strains with increased isobutanol biofuel tolerance, obtained from independent phenotype screening experiments. We prepared a total of ten libraries from these DNA samples, by pooling 3 strains each in eight libraries, and 4 strains each in the remaining two libraries. The ten libraries were each uniquely tagged, mixed, and sequenced as a single pool on an Illumina GA2x sequencer in single-end mode. This same mixture was sequenced on three replicate lanes, to assess the effect of different coverage levels on our results. The resulting approximately 90 million reads were filtered, aligned to the reference *E. coli* K-12 substr. MG1655 genome sequence, and scanned for sequence variants.

Among the three replicate lanes, each lane reported an average of 3988 SNPs, of which 3702 (92.8%) were called identically in all three lanes, 265 (6.6%) were called in two out of three lanes, and 21 (0.5%) were called in only one lane. We restricted our analysis to the 4099 high confidence single nucleotide polymorphism events that were called identically in at least two out of three lanes. Of these, 3596 mapped to 1808 *E. coli* annotated gene coding regions, including a total of 2379 non-synonymous SNPs in 1426 genes. The raw observations of these SNPs occurred at the expected frequency for a mutation in a single strain in a given library (i.e. approximately one-third of the reads covering that position in that library). An additional 23 mutations were reported at 100% allele frequency in all 10 libraries, and were identical in each of the ten libraries; these were excluded from subsequent analysis as mutations that were evidently present in the parent strain prior to mutagenesis. The 4099 SNPs showed a strong bias to occur at GC sites (GC/AT ratio of approximately 36), consistent with previous reports on NTG chemical mutagenesis [10]. Accordingly, we parameterized our calculations to take this bias into account (see Materials and Methods for details).

Two genes (*acrB* and *ydfJ*) were observed to be mutated in most of the strains, and several more were observed to be mutated in approximately a third of the strains (Table 1). Our p-value analysis (Table 2, 3) revealed a set of nine genes above the Bonferroni-corrected 95% confidence cutoff based on non-synonymous SNPs, two of them very strong (*acrB*, *marC*). Restricting the analysis to non-synonymous SNPs appeared to improve the p-value's significance several-fold. Consistent with the fact that the individual strains were generated in independent mutagenesis experiments, the mutations observed within a given gene were different in each library, except for four mutations in *acrB* that were each observed twice (at genomic positions 480611, 480674, 480931, 482319).

**Table 1:** Phenotype sequencing of 32 isobutanol tolerant *E. coli* strains (top 21 hits by raw SNP counts).

#SNP events	Genes
32	**acrB**
27	*ydfJ*
12	*cusA*, *entF*
11	*nfrA*, *prpE*
10	*febA*, *rhsD*, *sbcC*
9	*aesA*, *bscC*, **marC**, *mdlB*, *paoC*, *ykgC*, *yneO*
8	*ampH*, *kefA*, *yagX*, *ybaE*, *ybaL*

**Table 2:** Top 20 hits ranked by Bonferroni corrected p-value computed on all SNPs.

p-value	Genes	Description
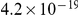	**acrB**	multidrug efflux system protein
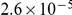	**marC**	inner membrane protein, UPF0056 family
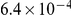	*aes*	acetyl esterase; GO:0016052 - carbohydrate catabolic process
0.0032	*ykgC*	predicted pyridine nucleotide-disulfide oxidoreductase
0.0035	*stfP*	e14 prophage; predicted protein
0.0095	*prpE*	Propionate–CoA ligase
0.032	*apt*	adenine phosphoribosyltransferase
0.039	*ampH*	penicillin-binding protein yaiH
0.052	*yihA*	GTP-binding protein required for normal cell division
0.053	*ispA*	geranyltranstransferase
0.060	*yceH*	conserved protein, UPF0502 family
0.13	*fepA*	iron-enterobactin outer membrane transporter
0.14	*cusA*	copper/silver efflux system, membrane component
0.15	*mdlB*	fused predicted multidrug transporter subunits of ABC superfamily: ATP-binding components
0.20	*ybbJ*	inner membrane protein that stimulates the ftsH htpX mutant suppressor activity of QmcA
0.30	*sfmH*	predicted fimbrial-like adhesin protein
0.33	*nfrA*	bacteriophage N4 receptor, outer membrane subunit
0.34	*yahB*	putative transcriptional regulator LYSR-type
0.40	*gsk*	inosine/guanosine kinase
0.40	*ybaE*	fused deaminase and uracil reductase

**Table 3:** Top 20 hits ranked by Bonferroni corrected p-value computed on non-synonymous SNPs.

p-value	Genes	Description
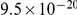	**acrB**	multidrug efflux system protein
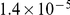	**marC**	inner membrane protein, UPF0056 family
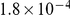	*stfP*	e14 prophage; predicted protein
0.0011	*ykgC*	predicted pyridine nucleotide-disulfide oxidoreductase
0.0035	*aes*	acetyl esterase; GO:0016052 - carbohydrate catabolic process
0.017	*ampH*	penicillin-binding protein yaiH
0.038	*paoC*	PaoABC aldehyde oxidoreductase, Moco-containing subunit
0.039	*nfrA*	bacteriophage N4 receptor, outer membrane subunit
0.044	*ydhB*	putative transcriptional regulator LYSR-type
0.12	*yaiP*	predicted glucosyltransferase
0.17	**acrA**	multidrug efflux system
0.25	*xanQ*	xanthine permease, putative transport; Not classified
0.25	*ykgD*	putative ARAC-type regulatory protein
0.35	*yegQ*	predicted peptidase
0.35	*yfjJ*	CP4-57 prophage; predicted protein
0.37	*yagX*	predicted aromatic compound dioxygenase
0.46	*pstA*	phosphate transporter subunit
0.48	*prpE*	propionate–CoA ligase
0.50	*mltF*	putative periplasmic binding transport protein, membrane-bound lytic transglycosylase F
0.63	*purE*	N5-carboxyaminoimidazole ribonucleotide mutase

Independent of this work, Atsumi et al. analyzed a single mutant strain SA481 with increased isobutanol tolerance, generated via growth in gradually escalating levels of isobutanol through 45 sequential transfers [15]. Sequencing of this mutant strain identified 25 IS10 insertions and a large deletion. Repair of each of these regions identified 5 genes as responsible for nearly all of the increased isobutanol tolerance in this strain: including *acrA*, *marC*; their data also indicated that *acrB* was inactivated in this strain. Atsumi et al. also validated the five genes' direct contribution to the phenotype by constructing individual and combination gene deletion strains.

Thus three of the top 20 genes identified by our phenotype sequencing analysis are experimentally validated as causing this phenotype. Others of our top scoring genes may also be real targets, but have not yet been tested via individual gene deletions. It is interesting that three pairs of genes appear to be from the same pathways: *acrA/acrB*, *ykgC/ykgD*, *yaiH(ampH)/yaiP*.

### Experimental Yield Analysis

**Figure 4:**
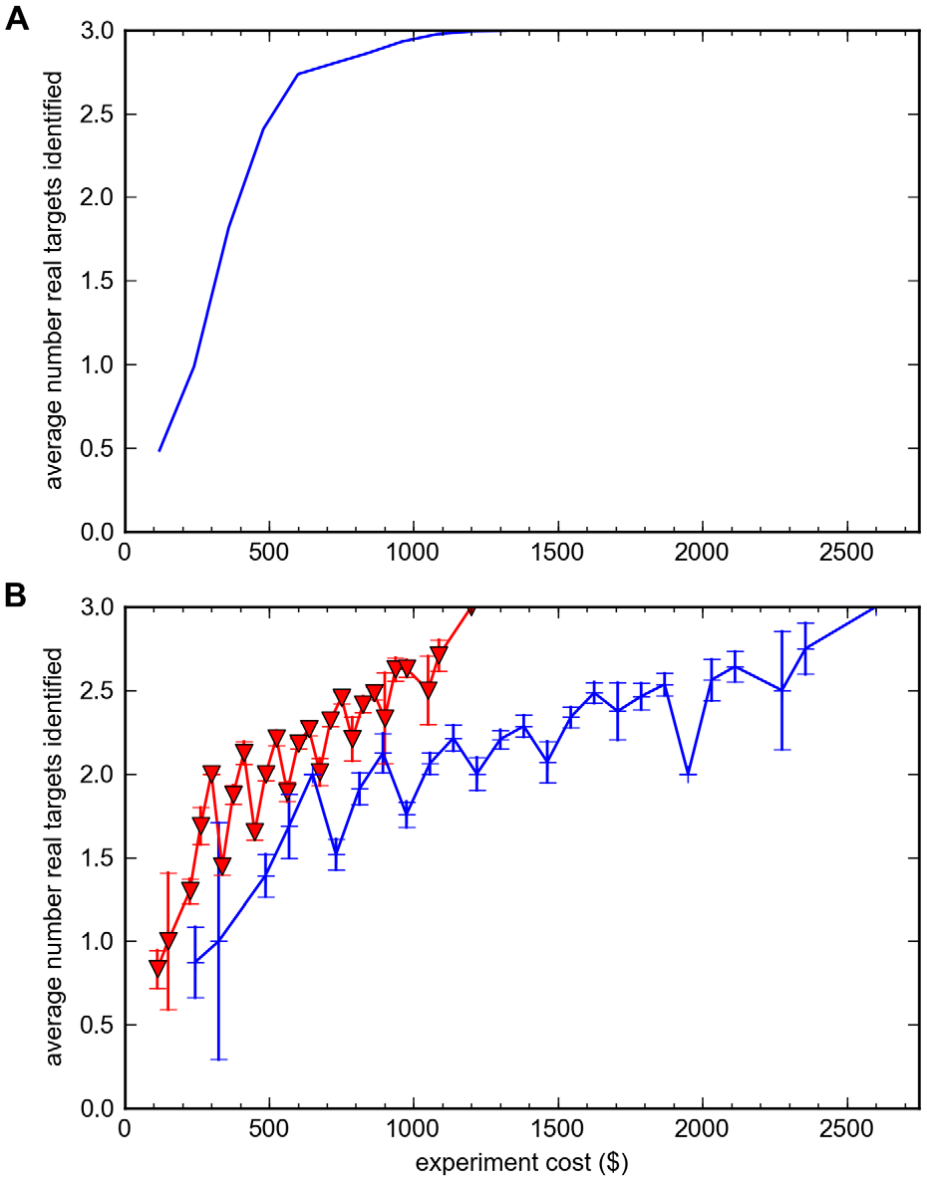
Modeled vs. experimental target gene yield as a function of increasing number of strains sequenced. **A**. Bioinformatic model of expected yield for discovery of 3 target genes, as a function of increasing number of strains sequenced, plotted vs. experiment cost, assuming one lane of sequencing at a cost of $37.50 per sequenced strain. **B**. Experimentally measured target gene discovery yields as a function of number of strains sequenced, plotted vs. experiment cost. Each data point is the average of all sub-experiments containing that number of strains; the error bar gives the standard error for this average from that set of sub-experiments. red line (inverted triangles): one lane of sequencing (32x coverage per library); blue line (+ signs): three lanes of sequencing (96x coverage per library, resulting in a total cost of $81.25 per strain).

Because our experiment was designed to split the 32 strains into 10 different tagged libraries (each containing 3–4 strains), it is possible to analyze the average true target gene discovery yield over all possible combinations of these 10 libraries, using the 10 separate tagged library datasets of reads. This constitutes a set of 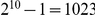 different possible experiments ranging in size from 3 to 32 sequenced strains. We ran our bioinformatic analysis separately on each of these 1023 experimental datasets to obtain the list of top 20 genes identified in each, and counted how many of the three validated true targets (*acrB*, *marC*, *acrA*) were identified. We consider one of these genes to be easy to discover (*acrB*, mutated in most strains), one somewhat harder (*marC*, mutated in a quarter of the strains), and the third hardest (*acrA*, mutated in less than a fifth of the strains). We then averaged the yields from different experiments that contained the same number of total strains. For example, eight different experiments contained just 3 strains; we averaged their yields. We plotted these average yield data (as a function of the number of strains sequenced) versus the total experiment cost (Fig. 4B), based on our actual reagent costs: $50 per library prep, and $700 per sequencing lane. For single lane sequencing, then, the cost per strain was 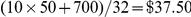.

These experimental data indicate that experiments costing $110–$150 (i.e. 3–4 strains) reliably identified one true target gene, and experiments costing $340–$525 detected two of the three target genes (Fig. 4B). In general, reliable detection of all three target genes was only obtained with the full set of 32 strains (total cost $1200). These results and costs were based on a single lane of sequencing with an average of 32x coverage per library. The added expense of triplicate sequencing (i.e. three lanes of sequencing yielding an average of 96x coverage per library) did not produce any significant increase in target gene discovery yields (Fig. 4B). These data indicate to us that at the level of pooling we used (3 to 4 strains per library), 32x coverage per library was adequate to obtain reliable detection of SNPs, so that the primary limiting factor for the target gene yield was simply the number of strains sequenced.

These experimental results match our bioinformatic model reasonably well (Fig. 4A). We modeled the expected target yield for a 3 target gene case, as a function of the number of strains sequenced, and plotted these yields against the experiment cost. The experimental data deviate from this model mainly in two respects: *acrB* appears to be considerably easier to find (mutated in most strains), whereas our model assumed an equal split among the three target genes (implying each would be found mutated in approximately a third of the strains); conversely, *acrA* appears to be harder to identify (mutated in less than a fifth of the strains).

### Bioinformatic Analysis of Experiment Optimization

To assess future prospects for improving phenotype sequencing, we considered a variety of factors. Since the success and yield of phenotype sequencing is limited fundamentally by the number of strains sequenced, the primary goal of phenotype sequencing design optimization is to maximize the number of strains that can be sequenced for a given experiment cost, i.e. to reduce the cost per strain. We used our bioinformatic model to analyze the effect of three different ways for achieving this: reducing the mutagenesis density; reducing the sequencing error rate; reducing the cost of sequencing.

**Figure 5:**
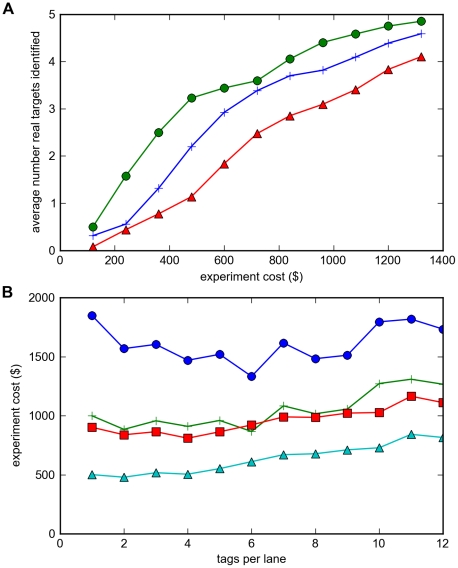
Effects of mutagenesis density, sequencing error, and sequencing cost on target yield and experiment cost. **A**. Average target discovery yield (y-axis) as a function of experiment cost (x-axis), at different mutagenesis densities: 20 mutations per genome (green circles); 50 mutations/genome (blue +); 100 mutations/genome (red triangles). **B**. Total experiment cost for analyzing 32 mutant strains (y-axis), as a function of the number of tagged libraries pooled per sequencing lane (x-axis), for different levels of sequencing error (1% vs. 0.1%) and different sequencing costs ($700 per lane vs. $350 per lane): 1% error, $700 per lane (blue circles); 0.1% error, $700 per lane (red squares); 1% error, $350 per lane (green +); 0.1% error, $350 per lane (cyan triangles).

As figure 5A shows, although reducing the mutagenesis density does not directly affect the cost of the sequencing experiment, it does increase the average yield of true targets discovered. Across a range of experiment sizes from 6 to 33 strains, reducing the mutagenesis density from 100 mutations per genome to 20 mutations/genome produced a target yield equivalent to that of sequencing approximately nine to twelve more strains, a cost savings of around $500. Given that the total cost of these experiments was $400–$1000, this is a dramatic improvement in yield per cost.

We next examined the effect of pooling different numbers of tagged libraries per sequencing lane, for a phenotype sequencing experiment of 32 strains. The number of tagged libraries per lane 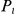 determines the effective coverage level per library; for the 4.6 Mb *E. coli* genome, the coverage per library is approximately 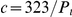. This in turn constrains the optimal number of strains that can be pooled per library (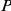), since increasing 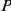 reduces the expected read count for a real mutation (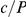) closer and closer to that expected for random sequencing error (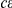), resulting in higher false positives and reduced target discovery yield. For each value of 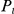 we determined the maximum value of 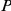 that maintained a high target discovery yield. Since the target discovery yield is primarily a function of the number of strains sequenced, the optimized yield was approximately the same for all the different tag-pooling values. Finally, we computed the total experiment cost, based on the number of tagged libraries that must be prepared (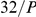) and the fractional number of sequencing lanes required to sequence all 32 strains.

These data show that at current costs ($700 per lane; $50 per library, October, 2010) and sequencing error rates (1%), the total experiment cost shows no clear trend as a function of the amount of tag-pooling 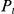 (Fig. 5B). At higher levels of tag-pooling (e.g. we used 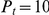 in our validation experiment), the reduced effective coverage per library means that only a smaller library-pooling factor can be used (e.g. we used 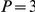 in our validation experiment). This results in higher library preparation costs, since the total number of libraries grows as 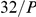. Conversely, as we reduce the tag-pooling factor 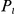, the effective coverage per library 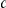 increases, allowing us to use a higher library-pooling factor 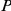. Unfortunately, the sequencing error rate 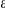 constrains how much we can increase 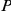, since the expected read count for real mutations (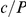) must be strongly distinguishable from that for sequencing errors (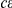). As a result, the total number of strains that can be sequenced per lane drops, and the resulting increase in sequencing cost offsets the reduced library preparation costs.

To assess future paths for improving phenotype sequencing yield and cost, we evaluated two different strategies: reduced sequencing error rate, and reduced sequencing cost per read. Improved sequencing approaches (such as multibase encoding schemes) may substantially reduce the sequencing error rate. We tested the effect of a ten-fold reduction in sequencing error (from 1% to 0.1%; Fig. 5B). This resulted in two effects. First, because this enabled pooling more strains per library (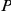), the total experiment cost was reduced by about $400–$800 across the range of tag-pooling levels. Second, a trend emerged for lower cost at lower tag-pooling levels. The reduced sequencing error rate made library preparation costs a dominant factor in the total cost; reduced tag-pooling enabled greatly increased library-pooling and dramatically reduced library preparation costs. We also tested the effect of reduced sequencing cost (reduced from $700 per lane to $350 per lane; increasing the number of reads per lane while keeping the cost per lane unchanged would have the same effect). Again, this had the effect of making library preparation costs the dominant factor in the total cost, resulting in a trend towards lower total cost at lower tag-pooling levels (Fig. 5B). Combining both strategies (reduced sequencing error rate and reduced sequencing cost) made this trend stronger: under these conditions, eliminating tag-pooling altogether (i.e. running a single library 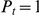 in each sequencing lane) reduced the total experiment cost by about a third relative to a high tag-pooling level (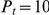), to a total experiment cost of about $500 (for sequencing 32 strains).

## Discussion

Taken together, these bioinformatic and experimental results suggest that phenotype sequencing can be a practical and effective method for identifying the genetic causes of a phenotype, provided several requirements are met: 1. a sufficient number of mutant strains with the desired phenotype, independently generated from a common ancestor, with a low density of random mutations; 2. a small enough genome (or region of genetic interest) to enable sequencing of this number of mutant strains at an acceptable cost; 3. a reference genome sequence that closely matches the ancestral genome, with gene annotations. We now discuss each of these requirements in turn.

The statistical power of phenotype sequencing depends entirely on the number of *independent* selection events (producing the same phenotype) that are sequenced. This can be achieved by performing independent mutagenesis experiments starting from a single parental strain, and screening each experiment for the desired phenotype. This both ensures that each mutant strain constitutes an independent mutation event, and permits control over the density of mutagenesis. Lowering the density of mutagenesis reduces the number of mutant strains that are needed to obtain a desired target gene discovery yield (but may also increase phenotype screening costs, due to the larger number of mutants that must be screened to find the desired phenotype).

Phenotype sequencing may also be applicable to mutant strains isolated from wild populations, tissue samples, or laboratory evolution under specific conditions [16] [17] [8]. Existing examples illustrate that it is possible to obtain a sufficient number of independent mutant strains from such sources [8]. However, naturally occurring mutant strains may require more costly sequencing analysis. Unless it is previously known that a given set of mutant strains form a star topology (i.e. their sequences are conditionally independent given the sequence of their most recent common ancestor (MRCA)), it would be necessary to reconstruct their detailed phylogeny, which is not possible using library-pooling. Instead, it would require a pure tag-pooling design, tagging each strain in a given lane uniquely, to obtain its individual sequence. In this case, target genes can be identified by calculating p-values based on the number of independent mutation events in each gene, inferred from the phylogenetic tree. Furthermore, we note that if a subset of mutant strains are believed to be conditionally independent given their MRCA, that subset can be pooled as a single library, reducing the cost without loss of information.

It should be emphasized that evidence of such phylogenetic structure (i.e. non-independence among mutant strains) can be easily detected even in library-pooled sequence data. Since independent mutation events are very unlikely to hit the exact same nucleotide site, each observed mutation should be found in only a single mutant strain. By contrast, if different strains share common ancestry subsequent to the MRCA (i.e. are not independent), by definition they will share some fraction of their mutations. Thus, detection of the exact same mutations in two or more strains constitutes a signature of non-independence. This can be detected either qualitatively, if the same mutations are separately detected in two different libraries, or quantitatively (if the two strains are in the same library, their shared mutations will be observed on average at double the expected read count). It should be noted that in some cases observation of the same mutation in two different strains might be due to *selection* (e.g. if a specific mutation is much more likely to cause the phenotype than other mutations are, or if only a small number of different mutations in the genome are capable of causing the phenotype), rather than due to common inheritance.

The cost of phenotype sequencing scales according to the size of the genome (or region of interest) being sequenced. Thus, it is clearly most useful for microbial and other small genomes. Increasing genome size proportionally increases not only the baseline sequencing cost, but also the genome-wide false positive rate due to sequencing error. This means that when the sequencing error rate per nucleotide site 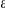 is held constant, a larger genome requires reducing the pooling factor 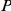 (in order to raise the mutation-call threshold enough to suppress false positives). This implies that for phenotype sequencing of larger genomes, it will be very valuable to reduce the per-nucleotide sequencing rate 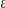, as discussed below.

Local variations in sequencing coverage might also raise the sequencing cost needed for obtaining a desired target discovery yield. Systematic studies of existing next-gen sequencing platforms have shown that they robustly detect >95% of SNPs despite local variations in coverage, with anomalously low coverage at approximately 0.1% (Illumina) to 1% (SOLiD) of nucleotide positions [18], especially AT-rich repeats. If poor coverage regions constitute only 5% of each gene region, they will not degrade target discovery yield significantly, since 95% of mutations in a target gene will still be detected. On the other hand, if a large fraction of each target gene fell into a poor coverage zone, that would reduce the target discovery yield proportionally. If an experiment gives poor discovery yield and suffers poor coverage across a large fraction of potential candidate genes, using a different sequencing platform would probably resolve the problem by supplying improved coverage in these regions (because the platforms differ markedly in their coverage biases [18]). However, existing data suggest that such problematic cases are likely to be uncommon.

To interpret the results of phenotype sequencing requires a reference genome sequence annotated with gene regions. Although it is possible to obtain results from phenotype sequencing without this, that would both require extra work, and dilute the biological meaningfulness of the results. First of all, it is not strictly necessary to have a reference genome sequence that exactly matches the actual parent of the mutant strains. Mismatches between the reference and the parent will simply be observed in each tagged library with an apparent allele frequency of 100%, and can be automatically excluded from consideration. For example, in our phenotype sequencing experiment we detected 23 mutations observed with 100% allele frequency in at least one library, and each such mutation was detected identically (at 100% frequency) in all ten libraries. We excluded these parental mutations from our analysis. Thus, the primary value of a reference genome sequence is that it greatly facilitates and accelerates phenotype sequencing, by enabling rapid alignment of reads and detection of mutations. In the absence of a reference genome, one would first have to assemble the reads *ab initio*, a considerably more complicated task. Similarly, accurate gene annotations with meaningful functional information are required not so much for obtaining phenotype sequencing results, but for biological interpretation of the results. In principle, for a completely unannotated genome, one could predict open reading frames (ORFs) and detect clustering of multiple mutations within individual ORFs just as effectively as with annotated gene regions. However, it might be harder to interpret the biological meaning of a discovered target gene, if little or no functional information could be found for it.

While phenotype sequencing can be useful for well-established microbial systems such as *E. coli*, it may have special value for genetically intractable organisms like *Chlamydia*, an important human pathogen. For example, in *Chlamydia*, researchers have identified a variety of potentially revealing mutant phenotypes, but deeper understanding of their genetic causes is impeded by the lack of powerful genetic systems for these bacteria [19]. For such organisms, phenotype sequencing can open up a fast path for directly identifying a phenotype's genetic causes, for any phenotype where a good screen exists for generating multiple independent mutant strains.

Our mathematical model of phenotype sequencing makes a number of assumptions that may be overly conservative relative to real-world phenotype sequencing experiments. We deliberately chose our model to represent the hardest possible case for phenotype sequencing, via the following conservative assumptions: 1. a maximum entropy split of the selection signal between all target genes; 2. only a single mutation is required to produce the phenotype; 3. a relatively high mutagenesis density and effective number of target genes. We now discuss each of these in turn. (We also note that while we only analyzed our experimental data for single nucleotide substitutions, in principle the same p-value scoring approach could be applied to other types of mutation events, e.g. deletions or insertions).

In our initial analysis, we assumed that each target gene is equally likely to be mutated, and equally likely to produce the phenotype. Both of these assumptions could be wrong. Splitting the selection signal equally among all target genes ensures that no target gene is any more detectable than any other target gene, and thus minimizes the detectability of the most detectable target gene. Introducing variability in either the probability of mutation or the probability of producing the desired phenotype *increases* the probability of detecting the top target gene. It seems unlikely that real-world phenotype sequencing targets will exactly match the hardest-case category. Many sources of gene variation are likely to create variability in the effective target size for a given phenotype. Empirically, we know that genes vary widely in size. We also expect that the contributions of different proteins to a given phenotype are likely to vary: whereas one protein might be absolutely central to that phenotype, such that a large fraction of amino acid mutations could cause the phenotype, in a protein that participates in only part of that function, perhaps only a small fraction of mutations could cause that specific phenotype. In our isobutanol tolerance mutants, we observed that one gene (*acrB*) showed a dramatically higher detectability than the other two validated targets (*marC, acrA*). Finally, whereas loss-of-function mutations may be possible in many genes within a pathway, gain-of-function mutations may be possible at only a subset of sites in a specific gene. Thus, a gain-of-function phenotype may display much stronger selection bias to a subset of target genes, making such target(s) easier to detect. Overall, we expect that real-world phenotype sequencing experiments will be easier (and more successful) than the estimates we have reported here from our uniform target size model.

We also assumed that the phenotype is produced via only a single mutational step from the parental strain. In other words, if a given mutant strain contains 100 mutations relative to the parent, we assume that only one of those mutations is causal (i.e. needs to be in a true target gene). This minimizes the “signal-to-noise” ratio (in this example, to just one causal mutation out of 100 total mutations), making the signal harder to detect. By contrast, if two or more mutations are required to produce the phenotype, that would multiply the signal-to-noise ratio proportionally, by two-fold or more. Our assumption of a single causal mutation means that the probability that each target gene is mutated in a given strain should sum to 1.0 (100%) over all the target genes. Empirically, in our isobutanol tolerance mutants we observed a target gene mutation probability sum much larger than 1.0: one gene (*acrB*) was itself mutated in nearly all the strains, and several more statistically significant genes were mutated in a third to a fifth of the strains each (*marC* and *acrA*, experimentally validated, plus *stfP, ykgC, aes*, not yet tested experimentally). Furthermore, Atsumi et al. have independently dissected the genetic causes in a single mutant strain, and found that five different mutations (in five genes) were responsible for the observed phenotype [15]. Similarly, Conrad et al. found that enhanced *E. coli* growth in lactate minimal media typically arose in each mutant strain via 5 to 8 contributory mutations in different genes [8]. Thus, we think that real-world phenotype sequencing experiments are likely to contain a higher signal-to-noise ratio than assumed by our single-causal-mutation model.

Our values for the mutagenesis density and total target gene number may also be larger than necessary. For example, our 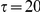 target gene model assumes that the selection signal is split equally over 20 genes, making each true target gene 20-fold harder to detect than turned out to actually be the case for *acrB* in our validation experiment. Are there really phenotypes in which 20 different genes can each cause the phenotype with equal probability? This seems like an extreme, difficult case, yet our results show that even it can be solved by sequencing a practical number of mutant strains (see Fig. 2C). Similarly, in our bioinformatic analyses and our experimental validation, we considered mutagenesis densities of greater than 100 mutations per strain. It should first be noted that such a density of potentially functional mutations (in our case, we restricted our analysis to non-synonymous mutations), corresponds to an even higher total mutation density (e.g. for 100 non-synonymous mutations, we might expect 150 total mutations). Since the experimenter can control the mutagenesis directly by reducing the concentration or time of mutagenesis, we suggest that future phenotype sequencing experiments should use a substantially lower mutagenesis density than we employed, to boost the signal-to-noise ratio.

Two additional trends appear to favor successful phenotype sequencing. First, the ongoing trend of decreasing sequencing cost per read (or equivalently, increased reads per unit cost) appears likely to continue for some time. We have sought to project the effect of this cost reduction on phenotype sequencing in Fig. 5B, which considers the effect of a two-fold reduction in sequencing cost. Second, sequencing technologies offer several ways to reduce the baseline sequencing error rate. For example, multibase encoding schemes can greatly increase the ability to distinguish real mutations from sequencing errors [20], assuming that the reference sequence is known. As shown in Fig. 5B, reducing the sequencing error rate to 0.1% has a similar effect on phenotype sequencing as reducing the sequencing cost two-fold.

To demonstrate the utility of phenotype sequencing, we have applied it to an important real-world problem in biofuels research, namely the production of long chain alcohols from well-characterized fermentation bacteria (in this case, *E. coli*). Recently, the UCLA-DOE Lab has engineered strains of *E. coli* that produce long-chain alcohols such as isobutanol and isopropanol [21] [22]. We believe phenotype sequencing brings several advantages to this work and to biofuels research in general: 1. It makes no assumptions about exactly what genes or pathways affect the yield, and can experimentally discover the factors that actually improve biofuel yield. 2. It utilizes the organism's own ability to evolve under externally applied selection pressure, to produce the desired result. 3. It employs an inexpensive, highly scalable technology (next-gen sequencing) to rapidly identify genes that actually cause the phenotype. In principle, this approach has an exciting ability to survey the factors that can improve yield of a desired biofuel.

## Materials and Methods

### A Mathematical Model of Phenotype Sequencing

To model independent phenotype selection events, we need probability distributions for the number of mutations in *target genes* (genes where mutations can cause the desired phenotype) and for non-target genes. First we consider a simple model in which genes are assumed to have uniform size, and then extend it to variable gene sizes. Note that we treat “size” as a general parameter combining the many factors that affect the probability of observing a mutation in a given region, including not only its length in the genomic sequence, but all other factors such as its base composition, mutational biases, and selection biases.

Independent mutations occurring over a genome are commonly modeled using the Poisson distribution. Specifically, if the expectation value for the number of mutations expected in a region is 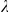, the probability of observing exactly 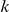 mutations in that region is given by 
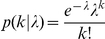



Now consider the following simple model of a phenotype selection screen. Assume that mutations at a subset of sites in the genome can cause the desired phenotype; call this the “target region” and designate its total size as 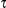. Defining the density of mutations resulting from mutagenesis as 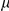, the expected number of mutations in the target region is 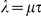. For convenience we express 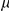 in terms of the number of mutations per gene, and 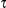 as simply the number of target genes. To model the effect of the phenotype selection screen, we require that at least one mutation be present in the target region, which alters the conditional mutation probability: 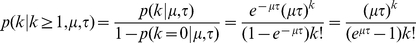



Thus, for a set of 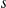 independent mutant strains that pass the phenotype screen, the distribution of the total number of mutations in the target region simply follows the sum of 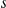 independent draws from this conditional distribution. We model this as follows: we extract the vector of values 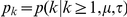 for a confidence interval 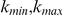 such that 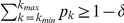 for a stringent confidence threshold 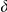, construct a multinomial distribution from this probability vector, and draw samples of 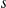 counts each from this multinomial. Specifically, each draw is a vector of 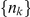 observation counts for each possible outcome 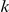, such that 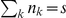. This yields a sample distribution for the total number of mutations 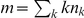 observed in the target region.

Given 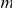 mutations in the target region, we model the distribution of mutation counts in individual target genes as follows. Assuming that there are 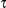 total genes in the target region, we construct a multinomial based on a probability vector of uniform gene probabilities 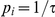, and draw a sample of 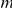 counts, i.e. a vector 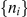 such that 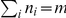. The 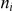 represent the individual mutation counts in each target gene. We then count the number of target genes 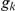 with a specified number of mutations 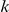. We sample their distribution by generating 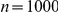 replicates of the above process, for any specific set of input parameters (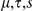, etc.).

We modeled the distribution of mutation counts in non-target genes by a similar methodology. If 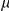 is the expected number of mutations per non-target gene in a single mutant strain, the distribution of total mutations per gene in 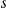 independent strains is itself just a Poisson with mean 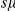: 
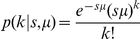



Again, we extract a probability vector of values 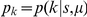 for a confidence interval 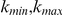 such that 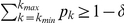, and construct a multinomial distribution from this probability vector. The distribution of the number of genes 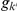 that contain exactly 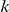 mutations is given by drawing 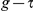 counts from this multinomial, where 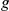 is the total number of genes in the genome, and 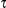 is the number of target genes in the genome.

We implemented these calculations in an open source Python module, *phenoseq*. Using optimized numerical libraries such as Numpy and Scipy [23], *phenoseq* can model over 600,000 mutant *E. coli* genomes per second on a single core of a 2.5 GHz Core 2 Duo CPU (early 2008 MacBook Pro). All of our code is available under an open source license at https://github.com/cjlee112/phenoseq.

#### Target Yield

We define the target yield as the number of true targets that can be discovered at a specified false discovery rate 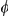. This is obtained by finding the smallest value 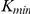 such that 
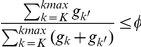
for all values of 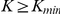, where 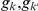 are respectively the number of true target genes with exactly 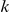 mutations, and the number of non-target genes with exactly 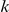 mutations. Then the yield 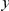 is just 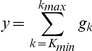



We computed average yields by sampling 1000 replicates of a given set of input parameters (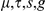, etc.).

#### Extension for non-uniform gene sizes

The above model can be extended for non-uniform target and non-target gene sizes as follows. We represent target size in terms of 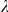, the mean number of expected mutations in a gene. For the multinomial representing target genes, instead of using a uniform probability 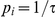 for each target gene, we instead compute each gene's target fraction based on its size 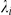: 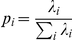



For non-target genes, we avoid the necessity of performing individual computations for all 4244 E. coli genes by subdividing the non-target genes into 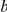 bins based on size. We sort the non-target genes by size, and assign each consecutive group of 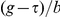 genes to a separate bin. Each bin is represented by the average size of the genes it contains. Then, instead of constructing a single Poisson for all non-target genes, we construct a separate Poisson representing the distribution of total mutations per gene in each bin with mean 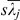, where 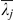 is the average gene size in bin 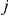. We then perform a separate multinomial calculation for each bin, and obtain the total number of genes 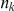 that contain exactly 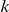 mutations simply by summing over the separate multinomials, i.e. 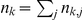, where the 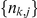 counts are drawn from the multinomial representing bin 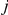.

Finally, we employ a simple definition of target size that takes into account mutational biases based on GC content. Specifically, we define a region's effective target size as: 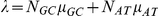
where 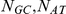 are the counts of GC vs. AT nucleotides in the region, and 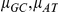 are the observed mutation probabilities per base at GC vs. AT nucleotides, measured genome-wide.

### Target Gene Candidate Scoring

To score the candidate genes, we first computed the p-value for a gene's observed mutation count 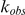 under the null hypothesis that it is not a target gene, based on its size 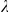: 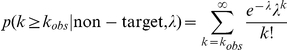



To apply this to multiple hypothesis tests (i.e. all the genes being analyzed) at some confidence level 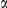, we applied the Bonferroni correction [24]: 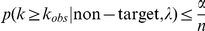
where 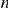 is the number of genes observed to be mutated at least once during the experiment. To apply this correction, we multiplied the p-value for each gene by the total number of genes being tested (e.g. for non-synonymous mutations, 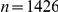 genes) to generate the corrected p-values shown in Tables 2 and 3.

To compute yields for models with variable gene size, we calculated the p-value for each gene, and sorted the genes by this value. We then found the largest cutoff value 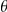 such that the fraction of non-targets out of all genes with p-value less than 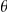 is less than the specified false discovery rate 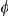. Then the yield 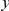 is the count of target genes with p-value less than 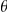.

### Analysis of Uniform vs. Variable Gene Size Models

**Figure 6:**
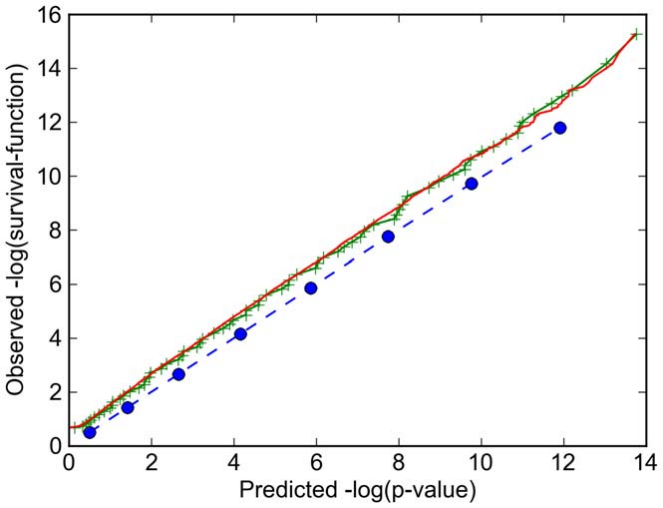
Effect of uniform vs. non-uniform gene size distributions on p-value scoring. Uniform gene-size model (blue circles, dashed line); Variable gene-size model based on subdividing the E. coli gene size distribution into ten size classes, each containing 424 genes represented by the average size within that class (green + markers); Variable gene-size model based on the exact sizes of all 4244 E coli genes (red line).

We directly tested the effects of uniform vs. non-uniform gene size models on the p-value scoring of non-target genes, using the following procedure to produce a plot of the expected negative log-survival-function (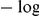 of the p-value) versus the actual observed negative log-survival-function (Fig. 6). We generated a sample of hit counts under our non-target model with our default assumptions (50 mutations/genome, 4244 genes, 80 strains sequenced, 1000 replicates). For each gene we drew a count 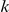 of how many times it was mutated in a sample of 80 strains, and calculated its p-value as described above. We converted these p-values to negative-log values. For 4244 genes times 1000 replicates, this gave about 4.2 million 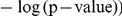 numbers. We then sorted these negative log values in ascending order (i.e. descending p-value). Finally we plotted each value against the negative log-survival-function of its true rank in this list, i.e. for list element 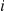, we plotted a datapoint (-logP[i], 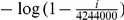), which should give a straight line on the 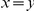 diagonal.

These data are shown in Fig. 6. For the uniform gene-size model, the p-values calculated by our scoring method matched exactly the actual survival-function observed in the sample. For the variable gene-size distribution of the actual sizes of all 4244 *E. coli* genes, the calculated p-value scores overall followed the actual survival-function observed in the sample (i.e. a linear plot in our graph), but were slightly shifted. Specifically, each p-value score (x-axis value) was actually observed at a slightly lower survival-function rank in the sample (y-axis value). (Since we plotted negative log values, this manifests as a slight upwards shift, to higher values on the y-axis).

These data indicate that our p-value scores have a slightly conservative bias versus their actual frequency of occurrence. Specifically, for calculations using the real, variable-length gene sizes, a given p-value score (say, 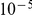) will actually occur among non-target genes at a *lower* frequency rank (approximately 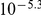). This means that a p-value score calculated using the real, variable-length gene sizes is slightly more significant than its value implies. Conversely, calculations using a uniform gene-size model (which lack this bias) will produce non-target gene p-values of a given significance strength *more* frequently than will actually occur in real-world calculations using real, variable-length gene data. That means that target gene discovery yields calculated using a uniform gene-size model will slightly underestimate the actual yield that will be obtained in real-world calculations using real, variable-length gene data. These data also show that approximating the exact gene size distribution with just ten size classes (green line in Fig. 6) yields almost identical results as the exact size distribution (red line in Fig. 6). On this basis, we used the ten size class approximation to compute Fig. 2, and the uniform size model to generate Figures 3, 4a, and 5.

As a minor technical point, we note that the slight upwards shift in Fig. 6 for variable gene-size models has a simple explanation. The p-value calculated for a single gene correctly predicts the frequency at which it will occur when it is mixed with other genes only if the other genes have exactly the same p-value distribution. This is not true if the genes differ in size. This error shifts the plot for variable gene-size models upwards beginning right at the origin of Fig. 6. Recall that the p-value for a given hit count 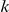 is simply one minus the sum of the probabilities of hit counts less than 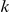; by definition, the p-value for 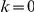 is exactly 1. Thus all occurrences of 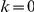 will be sorted (by descending p-value) earlier in the list than all 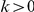 occurrences. They will then be followed by 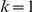 hits in very large genes, for which the probability of getting 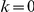 hits is near zero, and whose p-value is therefore close to 1. However, this large p-value is only valid for large genes, and doesn't take into account the fact that 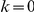 will occur *very* frequently among small genes. Consequently, this p-value's actual rank in the list will be pushed significantly down the list (by the many occurrences of 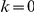 in small genes), relative to where it “should” be based on its p-value. This manifests in Fig. 6 as a vertical displacement right from the origin; this vertical displacement explains most of the shift across the entire line.

### Pooling and Sequencing Error Modeling

To model the effects of pooling and sequencing error on phenotype sequencing detection success rates, consider the average sequencing coverage 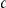 (average number of reads covering any given base), pooling factor 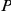 (number of strains pooled into a single tagged library), and sequencing error rate 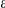 (defined here as the probability of erroneously observing a *specific* nucleotide, which is only a fraction of the *total* probability of observing any of the three incorrect nucleotides). If one strain in a pool contains a mutation at a specific site, the mutant nucleotide is expected to be present in a fraction 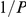 of the reads covering that site. We adopt the conservative assumption that the probability that this mutation will be called correctly in a given read is 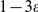. Assuming that reads are sampled independently from the different strains in the pool, the probability of fewer than 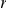 observation counts out of 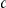 reads is drawn from a binomial with mean 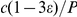: 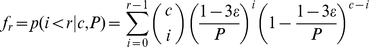



We will refer to 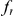 as the *mutation detection failure rate* associated with a detection threshold 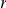.

Similarly, consider the probability of observing at least 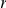 reads with a given erroneous nucleotide as drawn from a binomial with mean 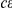: 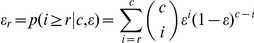



We refer to 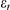 as the *mutation false positive rate* associated with a detection threshold 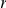.

We then modify the target and non-target gene modeling as follows. First, the multinomial probability vector representing the probability of assigning a mutation to each target gene is rescaled by 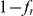:
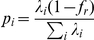
and an additional category with probability 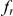 (representing *detection failure*) is appended to this vector. Counts drawn for this category from the multinomial are simply discarded. This models the process of occasionally failing to detect real mutations. Second, we draw counts of false positive mutation calls per target gene according to a Poisson with mean 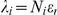, where 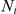 is the number of nucleotide sites in gene 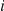. We simply add these counts to the vector 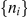 of true mutation counts per target gene to obtain the total “observed counts” per gene. For non-target genes, we simply adjust the effective “gene size” to reflect both false negative and false positive effects: 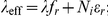
other aspects of the non-target gene calculation are performed identically. Note that the values of 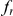 and 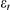 are based on the average coverage level 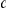. For our pooling model, we did not explicitly compute local variations in coverage, since deviations in regions with higher than average coverage will tend to cancel those in lower coverage regions, yielding overall values for 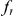 and 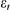 close to those computed from the average coverage level 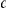.

### NTG mutagenesis and Isobutanol tolerance selection

Random mutagenesis was performed with N'-nitro-N-nitrosoguanidine (NTG) as previously described [25], using as a parent strain the *E. coli* JCL16 (BW25113/F' [traD36, proAB+, lacIq ZDM15]) strain described previously [26]. Briefly, exponential-phase cultures of JCL16 were concentrated two-fold by centrifugation and suspension in 0.1 M citrate buffer (pH 5.5) and exposed to N'-nitro-N-nitrosoguanidine (NTG) at a final concentration of 50 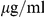 for 30 minutes at 37C to reach a percentage kill of approximately 50%. The cells were washed twice with 0.1 M phosphate buffer (pH 7.0) and grown in LB plus 4% glucose for two hours. The outgrown cultures were then challenged in the presence of 14 g/L isobutanol which is highly toxic to the wildtype type strain JCL16, for 12 h and plated on LB agar plates containing 25 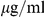 tetracycline. To select isobutanol tolerant mutants, isolated colonies were inoculated in 2 ml deep-96-well plates containing 300 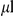 LB plus 8 g/L isobutanol per well and incubated for 24 h at 30C in a rotary shaker (250 rpm) (VWR). Bacterial growth was then determined by densitometry at 600 nm using a microplate reader (BioTek instruments Inc.).

### Library Preparation and Sequencing

Bacterial genomic DNA preparations from 32 strains were isolated using QIAamp DNA mini kit (Qiagen) with RNase treatment. Isolated genomic DNA was fragmented by sonication using Bioruptor (Diagenode) to an average size of 100–500 bp and confirmed by gel electrophoresis. 2 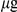 aliquots of fragmented genomic DNA from each strain were mixed to create genomic DNA pools. There were 10 pools created. Of these, 8 pools contained 3 strains and 2 pools contained 4 strains, such that each strain was in only one pool. 10 tagged genomic sequencing libraries were constructed using the Multiplex Sample Prep Oligo Kit following protocols provided by the manufacturer (Illumina). A mean library fragment size of 200–250 bp (mean insert size of 100–150 bp) was achieved by gel purification relative to standard sized markers. The purified library size distribution was confirmed by capillary electrophoresis in a Bioanalyzer (Agilent). 10 libraries were mixed in a proportion to maintain an equal amount of DNA from each strain in the multiplexed sequencing library. Final library concentration was determined by fluorescent based assay on the Qubit (Life Technologies). 7pmol of each mixed library was loaded onto each flow cell. The 76 base single end sequencing was carried out on a Genome Analyzer IIx (Illumina) within the UCLA DNA Microarray Facility using Single-Read Cluster Generation Kit v4 and Sequencing reagent v5. Base and quality calls were performed using RTA v1.8 (Illumina). All sequence data are being submitted to the NCBI Sequence Read Archive (SRA); accession numbers are pending.

### Sequencing Data Analysis

We used standard methods for sequencing read alignment and SNP detection. Each Illumina GA2x sequencing read file was first split into separate files for each of the ten unique prefix tags. Each file of tagged reads was aligned to the *E. coli* str. K-12 substr. MG1655 genome sequence (Genbank accession NC_000913), using the Novoalign software package (Novocraft, Selangor, Malaysia) in single-end read mode with default parameters. To analyze the alignments, we used the *samtools* software package [27] to convert the file to the BAM format, and then to the BCF format (binary encoding of the Genotype Likelihood format) via its **mpileup** command. We then ran the samtools program **bcftools** to search for single nucleotide polymorphisms and output them in VCF text format (for details, see http://samtools.sourceforge.net/mpileup.shtml). For our standard (3 lane) analysis, we filtered candidate SNPs by requiring a bcftools estimated allele frequency of 0.5 or less, and independent detection in two out three lanes; of these 4099 SNPs, 90.3% were independently detected in all three lanes. For our single-lane analyses, we only applied the allele frequency filter. It should be noted that a total of only 62 additional SNPs were detected in only one out of three lanes; this constituted only 0.5% of the SNPs detected by each individual lane. Further analysis of these data were performed using our own code written in Python, which mapped the SNPs to annotated genes (based on the CDS annotations for Genbank accession NC_000913.2); determined the specific amino acid substitution associated with each SNP; and computed p-value scores for each gene as described above. We used the Biopython module [28] to read the Genbank annotation data, and the Pygr module [29] to map SNPs to the CDS annotations and determine their associated amino acid substitutions. We used statistical functions from the scipy.stats module [23] as part of computing p-values. All of our code is available under an open source license at https://github.com/cjlee112/phenoseq.
